# Allograft Inflammatory Factor 1 Functions as a Pro-Inflammatory Cytokine in the Oyster, *Crassostrea ariakensis*

**DOI:** 10.1371/journal.pone.0095859

**Published:** 2014-04-23

**Authors:** Ting Xu, Jiasong Xie, Baojian Zhu, Xiao Liu, Xinzhong Wu

**Affiliations:** 1 Laboratory of Marine Life Science and Technology, College of Animal Sciences, Zhejiang University, Hangzhou, Zhejiang, China; 2 Zhejiang institute of freshwater fishery, Huzhou, Zhejiang, China; 3 College of Life Science, Anhui Agricultural University, Hefei, Anhui, China; 4 Ningbo University, Ningbo, Zhejiang, China; University of Nebraska Medical center, United States of America

## Abstract

The oyster *Crassostrea ariakensis* is an economically important bivalve species in China, unfortunately it has suffered severe mortalities in recent years caused by rickettsia-like organism (RLO) infection. Prevention and control of this disease is a priority for the development of oyster aquaculture. Allograft inflammatory factor-1 (AIF-1) was identified as a modulator of the immune response during macrophage activation and a key gene in host immune defense reaction and inflammatory response. Therefore we investigated the functions of *C. ariakensis* AIF-1 (Ca-AIF1) and its antibody (anti-CaAIF1) in oyster RLO/LPS-induced disease and inflammation. Ca-AIF1 encodes a 149 amino acid protein containing two typical Ca^2+^ binding EF-hand motifs and shares a 48–95% amino acid sequence identity with other animal AIF-1s. Tissue-specific expression analysis indicates that Ca-AIF1 is highly expressed in hemocytes. Significant and continuous up-regulation of Ca-AIF1 is detected when hemocytes are stimulated with RLO/LPS (RLO or LPS). Treatment with recombinant Ca-AIF1 protein significantly up-regulates the expression levels of LITAF, MyD88 and TGFβ. When anti-CaAIF1 antibody is added to RLO/LPS-challenged hemocyte monolayers, a significant reduction of RLO/LPS-induced LITAF is observed at 1.5–12 h after treatment, suggesting that interference with Ca-AIF1 can suppress the inflammatory response. Furthermore, flow cytometric analysis indicated that anti-CaAIF1 administration reduces RLO/LPS-induced apoptosis and necrosis rates of hemocytes. Collectively these findings suggest that Ca-AIF1 functions as a pro-inflammatory cytokine in the oyster immune response and is a potential target for controlling RLO infection and LPS-induced inflammation.

## Introduction

Invertebrates lack adaptive immunity, but their immune systems are capable of effective immune responses and are complex and diverse [1], [2]. Although much has been learned from the *Drosophila* model system, we have not yet achieved a comprehensive view of innate immunity across the broad spectrum of invertebrate phyla [3], [4], [5]. Mollusca is one of the most diverse groups of invertebrates with more than 100,000 living species mostly inhabiting marine environments [6]. Models from this prominent invertebrate phylum provide important opportunities to study innate immunity and could provide insight on the evolution of the immune system. Advances in genomic technology now allow for progress in this area. Here, we focus our studies on this prominent phylum using a representative bivalve species, the oyster *Crassostrea ariakensis* as a model organism.

*C. ariakensis* is one of the most economically important oysters cultivated in southeastern China, especially in Guangxi, Guangdong and Fujian provinces. Aquaculture of this species has suffered from massive infection and severe mortalities caused by the pathogen rickettsia-like organism (RLO) [7], [8]. Rickettsias are Gram-negative bacteria, generally described as obligate intracellular pathogens that multiply only within host cells [9]. This prokaryote has been reported in many aquatic species including fishes [10], [11], crustaceans [12], [13] and mollusks [14], [15]. In marine mollusks, more than 25 species have been reported worldwide to be infected with RLOs, resulting in mortalities and dramatic economic losses [25]. Prevention and control of this disease is becoming a priority for the further development of oyster aquaculture.

In our team previous studies, we have made great efforts to solve this problem. We have purified RLOs directly from infected oyster tissues following the differential speed centrifugation and renografin density gradient centrifugation method that we developed [16]. The gene *ompR*, which encodes an outer membrane protein of RLO has been identified and we have characterized its role in promoting the immune response by analyzing the interaction between RLO *ompR* and the oyster immune system [17]. In the course of this work we have made some progress in characterizing the oyster defense system. The oyster transcription factor cAMP response element-binding protein (CREB) [18], the oyster Tolloid-like gene [19] and a tetraspanin family member gene [20] are involved in oyster immune responses against infection, and a novel homolog of human soluble TRAIL (TNF related apoptosis-inducing ligand), oyster sTRAIL, can suppress RLO infection through activation of p38-MAPK pathway [21]. Recently, we constructed a RLO-challenged oyster hemocyte cDNA library [22], from which a high mobility group box (HMGB) homolog Ca-HMGB was identified and found to function as a pro-inflammatory cytokine in oyster immune reaction [23].

Here, we reported another protein identified from the hemocyte cDNA library, a novel allograft inflammatory factor 1 (AIF-1), which we named Ca-AIF1. AIF-1 is a cytoplasmic, 17 kDa interferon-γ-inducible calcium-binding EF-hand protein. It was demonstrated to be a modulator of the immune response during macrophage activation in mammals [24], [25]. Also, evidence suggests that AIF-1 plays a significant role in different host response to inflammatory stimuli and to the wider host immune defense reaction [26], [27].

Here we analyze the expressions of a series of obtained immune-related genes LITAF, MyD88 and TGFβ of oyster *C. ariakensis* in order to characterize the potential pro-inflammatory cytokine function of Ca-AIF1 using prepared recombinant Ca-AIF1 protein and its polyclonal antibody. The ability of the anti-CaAIF1 antibody treatment to reduce the oyster inflammatory reaction and to reduce hemocyte apoptosis and necrosis upon exposure to RLO/LPS challenge were also analyzed.

## Materials and Methods

The supporting CONSORT checklist is available as supporting information; see Checklist S1.

### Ethics statement

The oyster *C. ariakensis* is one of the most widely consumed seafoods and therefore an economically important marine species in China and around the world. All the experiments were conducted strictly according to the regulations of Chinese local and central governments. The treatment of experimental animals complied with the Laboratory Animal Law in China. The oysters used in this study were bought from the fishermen who cultivated the oysters in Guangxi province. The field studies did not involve endangered or protected species and no specific permits were required in this field study in China. In the course of the experiment, we have made our best effort to minimize animal suffering.

### Oyster experimentation and tissue isolation

Healthy oysters aged 2–3 years old were obtained from Qinzhou bay (Guangxi, China) and maintained in artificial seawater with a cycling system at 19±1°C for one week before experiments were carried out. The individual size was about 8.1*5.3*11.6 (length*width*height) on average. Three untreated oysters were randomly sampled for hemocytes, gill, mantle, digestive gland, gonads and adductor muscle tissue in order to characterize tissue-specific Ca-AIF1 expression. Total RNA extraction was performed immediately after dissection using the RNA_fast1000_ purification kit (Feijie, China) according to kit procedures. Total RNA isolated from each organ was reverse transcribed into cDNA with M-MLV RTase cDNA Synthesis Kit (Takara, Japan) following the kit instructions and stored at −20°C.

### RLO preparation

RLOs were prepared as reported previously [22]. Moribund oysters sampled from Qinzhou bay (Guangxi, China) were washed with PBS (phosphate-buffered saline, pH 7.4), and the isolation and purification of RLOs were carried out following the ‘differential speed centrifugation and renografin density gradient centrifugation’ method established by Li and Wu [16]. After that, purified RLOs were cultured using the ‘chick embryo culture’ method (unpublished data), then were collected and centrifuged at 12,000×g for 20 min, and finally resuspended in sterile seawater (OD_600_ = 1.151) and stored at −80°C.

### Sequence characterization and phylogenetic analysis of Ca-AIF1

Ca-AIF1 clones containing full open reading frame (ORF) were identified from a previously constructed cDNA library [22]. Homology search was carried out using the BLAST program against the GenBank database at NCBI (http://www.ncbi.nlm.nih.gov/BLAST). ORF was acquired with ORF Finder tool (http://www.ncbi.nlm.nih.gov/gorf/). Domains/motifs were identified using the PROSITE profile database (http://cn.expasy.org/prosite). Multiple sequence alignment was performed using ClustalW program version 1.8. A phylogenetic tree was constructed using the neighbor-joining method with MEGA 3.1 package and the reliability of the tree was estimated via bootstrap analysis with 1000 replicates.

### Protein expression, purification and polyclonal antibody production

Based on the full sequence of Ca-AIF1 ORF, specific PCR primers were designed with restriction enzyme sites (*Bam*H I and *Xho* I) added to the gene sequence (Table 1). PCR was carried out using reverse transcribed cDNA from hemocytes as the template and performed under the following conditions: initial denaturation at 94°C for 5 min; followed by 35 amplification cycles (94°C for 30sec, 53°C for 30 sec, 72°C for 45 sec) and a final elongation step at 72°C for 10 min. PCR products purified by agarose gel electrophoresis were digested with *Bam*H I and *Xho* I (Takara, Japan) and ligated to a pET-32a expression vector (digested with the same restriction enzyme) (Novagen, Germany). The recombinant plasmids (pET-CaAIF1) were identified by sequencing and then transformed into competent BL21 (DE3) cells (Novagen, Germany). The cells were cultured in a shaken incubator at 230 rpm, 37°C in the presence of ampicillin (100 µg/ml) to OD_600_ values of 0.4∼0.6 and then were induced by 0.5 mM isopropyl-1-thio-β-D-galactopyranoside (IPTG, Sigma, USA) while shaken at 230 rpm, 30°C for 6 h. Cells were then collected by centrifugation at 10,000×g for 10 min and recombinant fusion protein was purified by affinity chromatography using His Bind Purification Kit (Novagen, Germany) following the manufacturer's protocol. Ca-AIF1 polyclonal antibody was prepared as discussed previously [18]. In brief, purified protein was homogenized in complete Freund's adjuvant (Sigma, USA) and two New Zealand White rabbits were immunized (2 mg of purified proteins for each rabbit) three times at 2-week interval. A booster injection was given after another week with 1 mg of purified protein in incomplete Freund's adjuvant. Rabbit serum was collected ten days after the last immunization and stored at −80°C.

**Table 1:** Sequences of primer pairs used in this study.

Putative Gene	Primer Sequence (5′-3′)
Ca-AIF1 ORF primer F	TTGGATCCATGTCGGTGGACTTCAAAG
Ca-AIF1 ORF primer R	CTCTCGAGCTATGGCAGATCGGAGAG
28s real-time RT-PCR primer F	GAATCCCTCATCCTAGCGA
28s real-time RT-PCR primer R	CACGTACTCTTGAACTCTCTC
LITAF real-time RT-PCR primer F	GAGAAGTCAGGACCCCA
LITAF real-time RT-PCR primer R	TTAGATTCTGTCGTAGCG
MyD88 real-time RT-PCR primer R	CAGGAGTTCGTCAGTCTC
MyD88 real-time RT-PCR primer F	GACTCTCAGCTCTTTCTTG
TGFβ real-time RT-PCR primer R	GAGAATAGTGGTCTGGTGAT
TGFβ real-time RT-PCR primer F	CAGGCTGCATGAACGACTG
AIF1 real-time RT-PCR primer R	GGCAAAGACCCATCTAGAGC
AIF1 real-time RT-PCR primer F	CTGCGGGTTTAGCTTTCTCT

Restriction enzyme sequences were underlined.

### Hemocyte monolayer preparation and immune challenge

Hemocyte monolayers were prepared as described previously [18], [22] according to Lacoste et al. [28] and Canesi et al. [29]. Briefly, hemolymph was extracted from the pericardial cavity of ten oysters for each experiment, pooled to about 15 ml volume and adjusted to 10^6^ cells/ml by addition of Hank's balanced salt solution (HBSS, adjusted to ambient seawater salinity). Hemolymph serum was obtained by centrifugation at 800×g for 5 min and supernatant was sterilized through a 0.22 µm filter. Hemolymph (1 ml) was dispensed into sterile Petri dishes and incubated at 15°C for 30 min. Non-adherent hemocytes were carefully rinsed with HBSS, and 1 ml of above filtered hemolymph serum containing penicillin G (50 units/ml) and streptomycin (50 µg/ml) was added to the hemocyte monolayer and kept at 15°C before use.

Twenty-four hours after the hemocyte monolayers were seeded, the hemolymph serum was replaced with fresh serum also containing penicillin G and streptomycin. Then the hemocyte monolayer was treated as follows: (1) hemocytes were incubated with RLOs prepared as before (RLO OD_600_ = 1.15, final concentration, 1 µl RLO/10^6^ hemocytes); (2) hemocytes were incubated with LPS (final concentration,100 ng/ml); (3) hemocytes were incubated with purified recombinant Ca-AIF1 proteins (final concentration, 1 µg/ml); (4) hemocytes were incubated with RLOs (final concentration, 1 µl RLO/10^6^ hemocytes) and Ca-AIF1 polyclonal antisera were produced (1∶1000); (5) hemocytes were incubated with LPS(final concentration,100 ng/ml)and Ca-AIF1 polyclonal antisera (1∶1000); (6) Hemocytes were incubated with filtered seawater. All treatments were carried out over five different time intervals (0 h, 1.5 h, 4 h, 8 h and 12 h). Total RNA from each set of treated hemocytes was then extracted and reverse transcribed as described above.

### Western blotting

Western blot analysis was used to validate the specificity of the prepared Ca-AIF1 antibody. Recombinant Ca-AIF1 protein was separated on a 12% SDS-PAGE gel and transferred onto a polyvinylidene difluoride (PVDF) membrane (Millipore, USA) using an electrophoretic transfer system (Bio-Rad, USA). Then the membranes were blocked with PBST (PBS pH 7.4, containing 0.1% Tween-20) containing 5% skim milk for 2 h at room temperature, and probed with prepared Ca-AIF1 antibody or pre-immunized rabbit serum (1: 5000 diluted in blocking buffer) for 1 h at room temperature. After washing three times with PBST, membranes were incubated with horseradish peroxidase (HRP)-conjugated sheep anti-rabbit IgG antibody (Dingguo, China, 1∶2000 diluted in blocking buffer) for 1 h at room temperature. The immune complexes were detected with an HRP-DAB Detection Kit (Tiangen, China).

### Quantitative RT-PCR

Quantitative RT-PCR was performed to analyze the following: (1) tissue-specific expression of Ca-AIF1 in untreated oysters; (2) Ca-AIF1 mRNA expression profile with RLO/LPS incubation; (3) mRNA expression profile of LITAF (LPS-induced TNFα factor, GenBank No:EU249541), TGFβ (transforming growth factor-beta, Genbank No:EU249542) and MyD88 (myeloid differentiation primary response protein 88, GenBank No:EF221769) with the Ca-AIF1 incubation; (4) mRNA expression profile of LITAF with RLO/LPS added/not added with Ca-AIF1 polyclonal antiserum incubation.

Gene-specific primers were designed using Primer 5.0 software based on the obtained sequence and reported sequences while the 28SrDNA gene (GenBank No: AF137052) was used as an expression standard (Table 1). The real-time RT-PCR was performed with a SYBR Premix Ex Taq Kit (Takara, Japan) in an iCycler iQ thermocycler (Bio-Rad) using the following procedure: initial denaturation at 95°C for 3 min; followed by 40 cycles of amplification (95°C for 20 sec and 55°C for 40 sec).

### Statistical analysis

The relative expression level of each gene was determined by the Livak 2^−ΔΔCT^ method [30], [31] and the values were presented as mean ± SEM of independent experiments done in triplicates. Before using this method, we verified that PCR efficiency of reference and target genes were approximately equal and 28SrDNA gene was used as endogenous control.

For tissue specific expression, the calculated relative expression level of Ca-AIF1 was compared to the mRNA expression level in the adductor muscle and the data were analyzed by Student's t-test. Differences were considered statistically significant when p values were less than 0.05.

For other challenged samples, the gene expression level at each time point was relative to the seawater-challenged samples and the data were subjected to a one-way analysis of variance (ANOVA) followed by Duncan's Multiple Range test using the SPSS program. Differences were considered statistically significant when p values were less than 0.05.

### Flow cytometry analysis

The hemocyte monolayer was cultured as described above. 24 h after seeding, we changed the hemolymph serum (containing penicillin G and streptomycin) and replaced it with fresh hemolymph serum which also contained antibiotics. Then the hemocyte monolayer was treated as follows: (1) hemocytes were incubated with RLO/LPS (final concentration, 1 µl RLO/10^6^ hemocytes; LPS: 100 ng/ml); (2) hemocytes were incubated with RLO/LPS and Ca-AIF1 polyclonal antiserum (1∶1000); (3) hemocytes were incubated with RLO/LPS and pre-immune serum (1∶1000). Twelve hours after treatment, flow cytometry analysis was performed to detect apoptotic and necrotic cells using an Annexin V/PI apoptosis kit (MultiSciences Biotech, China). According to the kit instruction, hemocytes (about 5×10^5^ cells) were collected by centrifuging 500×g for 15 min, resuspended in Annexin-binding buffer with subsequent addition of Annexin V-FITC and Propidum Iodide (PI), and then incubated in the dark for 15 min at room temperature. The data were then analyzed using a FACSCalibur (Becton Dickinson, USA) with CellQuest Software (Becton Dickinson). FITC and PI-fluorescence were collected to distinguish among living cells, early apoptotic cell, late apoptotic cell and necrotic cells.

## Results

### Sequence analysis of Ca-AIF1

**Figure 1:**
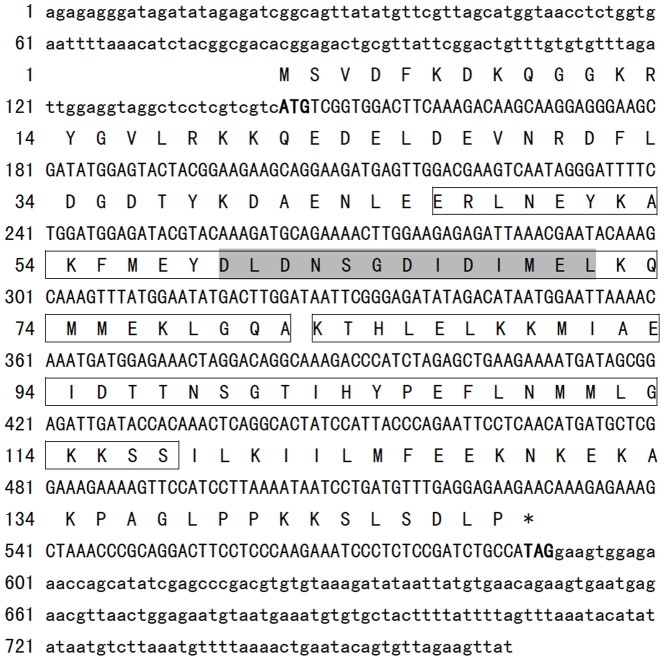
Nucleotide and deduced amino acid sequences of Ca-AIF1 from the oyster, *Crassostrea ariakensis*. The ORFs of the nucleotide sequences and deduced amino acid are shown in upper-case letters, the 5′- and 3′-UTRs are shown in lower-case letters. Nucleotides and amino acids are numbered on the left of the sequences. The initiation codons and stop codons are in bold. The boxed area indicates the region of EF-hand-like motif (with the conserved loop segment shaded).

A 766 bp cDNA sequence (GenBank No. HM749971) was obtained by screening the oyster cDNA library we constructed previously [22]. It contains an ORF of 447 bp encoding 149 amino acid residues with a predicted molecular weight of 17.13 kDa and a theoretical isoelectric point (*p*I) of 5.308. The 5′ and 3′ untranslated regions (UTR) contain 143 bp and 176 bp, respectively. Blastp analysis of the deduced amino acid sequence revealed that it is a homologue of AIF-1 protein similar to that identified in other species. We named it Ca-AIF1. The sequence and its deduced amino acid sequence are shown in Figure 1.

Two EF-hand calcium-binding motifs were found in the amino acid sequence ranging from residues 46–81 and 82–117, respectively by the PROSITE program. Additionally, several potential biologically active site motifs were identified including two amidation sites (residues 10–13 and 112–115), one tyrosine kinase phosphorylation site (residues 30–38), one protein kinase C phosphorylation site (residues 37–39), three casein kinase II phosphorylation sites (residues 37–40, 83–86 and 143–146), one N-myristoylation site (residues 79–84) and one cAMP-/cGMP-dependent protein kinase phosphorylation site (residues 114–117). The signal peptide and nuclear localization signal were absent within the deduced amino acids of Ca-AIF1 (Figure 1).

**Figure 2:**
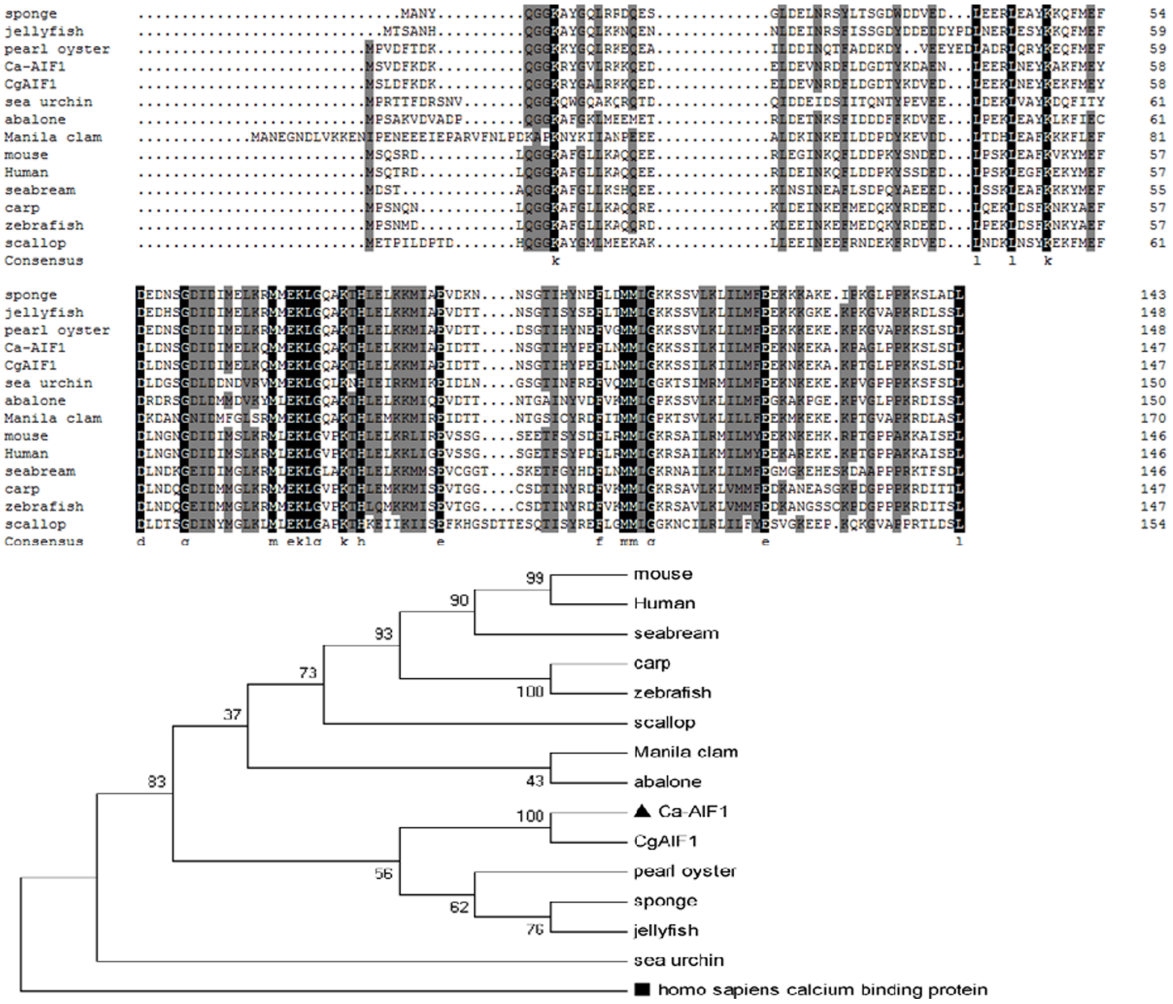
Alignment and phylogenetic analysis of Ca-AIF1 amino acid sequences with other animal AIF-1s. (A) Multiple alignment. Conserved amino acids were shaded and each shade represents a degree of conservation (black, 100%, grey, 70%). The alignment was taken by ClustalX program. (B) Phylogenetic analysis was performed by MEGA (version 3.1) program based on the AIF-1 amino acid sequences from various animals. The phylogenetic tree was constructed using neighbor-joining method and bootstrap 1000. The human calcium binding protein was taken as out-group root.

Multiple alignment of Ca-AIF1 with other AIF-1 proteins from various animals (including invertebrates and vertebrates) showed amino acid sequence identity ranging from 48%–95% (Figure 2A and Table 2). A phylogenetic tree was generated based on multiple alignment using the neighbor-joining method rooted with the human calcium binding protein (GenBank No. AAC27697). The results show that Ca-AIF1 was branched with *Crassostrea gigas* (GenBank No. EKC34896) and clustered to, *Pinctada martensi* (GenBank No. AEX55297), *Suberites domuncula* (GenBank No. CAC38780) and an uncultured cnidarian (GenBank No. ABA42882) (Figure 2B). The sequences used for multiple alignment and phylogenic analysis and their percentage identity with Ca-AIF1 are shown in Table 2.

**Table 2:** Sequences used for multiple alignment and phylogenic analysis and their percentage identity with Ca-AIF1.

Species	Common name	Accession No	Identity %
***Crassostrea ariakensis***	Ca-AIF1	AEJ08752	100
***Crassostrea gigas***	CgAIF1	EKC34896	95
***Pinctada martens***	Pearl oyster	AEX55297	74
***Suberites domuncula***	Sponge	CAC38780	72
***Uncultured cnidarian***	Jellyfish	ABA42882	68
***Haliotis discus discus***	Abalone	ACJ65689	59
***Homo sapiens***	Human	AAB05003	55
***Sterechinus neumayeri***	Sea urchin	ACO40483	53
***Pagrus major***	Seabream	BAA36938	52
***Mus musculus***	Mouse	BAA28216	52
***Cyprinus carpio***	Carp	BAA32796	50
***Ruditapes philippinarum***	Manila clam	ACU83234	49
***Danio rerio***	Zebrafish	NP_942571	48
***Chlamys farreri***	Scallop	ABY84846	48

### Tissue-specific expression of Ca-AIF1

**Figure 3:**
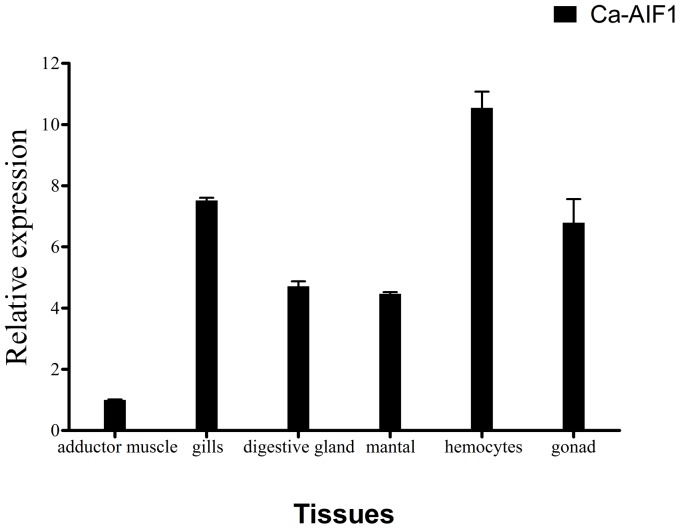
Expression levels of Ca-AIF1 were detected in various tissues by real-time RT-PCR. The data were normalized by mRNA expression in the adductor muscle and 28SrDNA gene was used as endogenous control. The values were presented as mean ± SEM of independent experiments done in triplicates and analyzed by Student's t-test. *p≤0.05 when compared to control value.

The expression levels of Ca-AIF1 in various tissues including hemocyte, gills, mantle, digestive glands, gonads and adductor muscle were investigated using real-time RT-PCR. Specificity of primer pairs was verified by sequencing the PCR product before use and level of expression in adductor muscle was used to normalize the expression of other tissues. The results show that Ca-AIF1 transcripts are ubiquitously expressed in all examined unstimulated tissues and that the highest and lowest expression was detected in hemocyte and adductor muscle, respectively (Figure 3).

### Protein expression, purification and polyclonal antibody production

**Figure 4:**
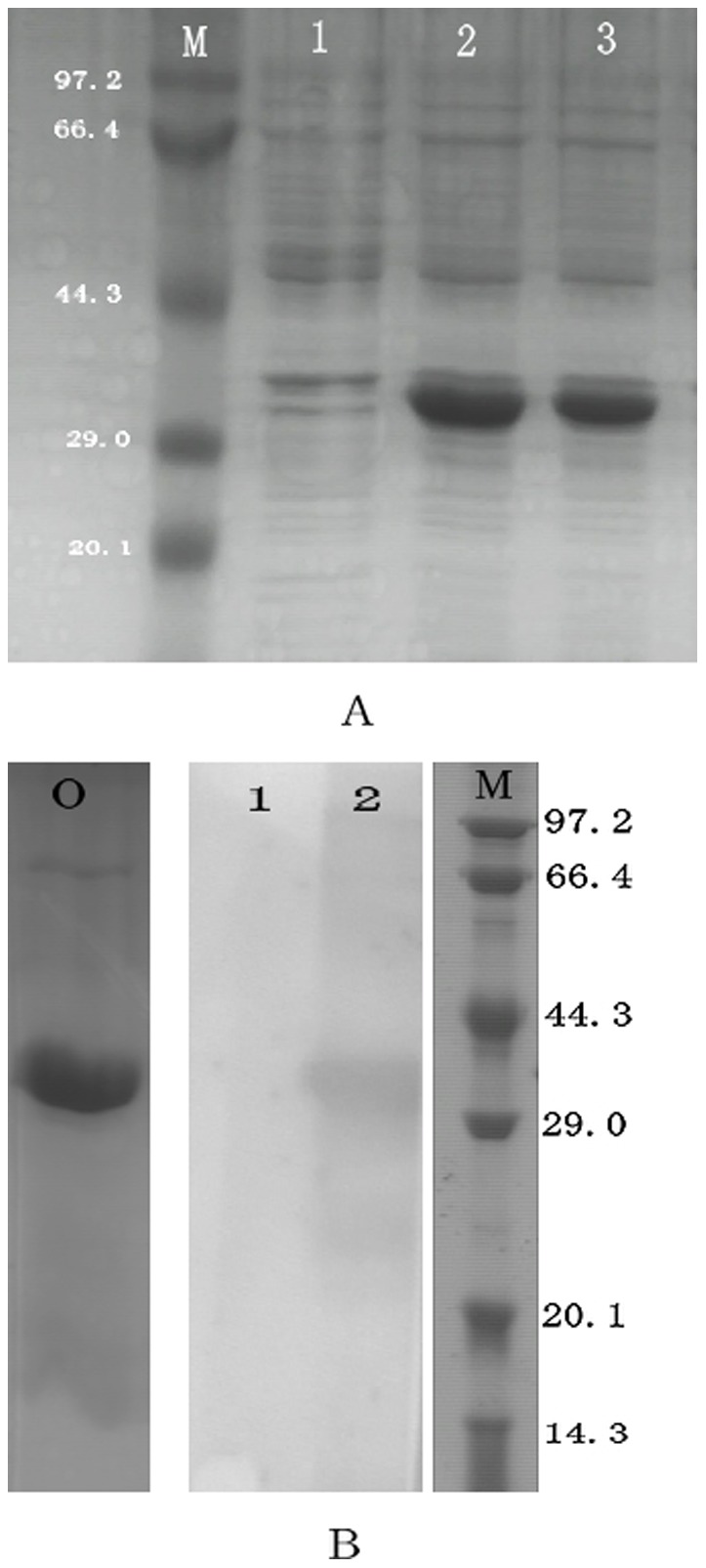
SDS-PAGE and western blotting analysis of Ca-AIF1. (A) SDS-PAGE analysis of pET-32a-Ca-AIF1 expressed in *E.coli* BL21 (DE3). M, Protein mark; 1, No induction; 2, 0.5 mM IPTG induction; 3, 1.0 mM IPTG induction. (B) SDS-PAGE analysis of purified Ca-AIF1 and western blotting analysis of anti-CaAIF1 (1∶5000). 0, SDS-PAGE analysis of purified Ca-AIF1; 1, Western blotting analysis of pre-immunized rabbit serum; 2, Western blotting analysis of anti-CaAIF1; M, Protein mark.

The complete ORF of Ca-AIF1 was cloned into the pET32a expression vector. A protein with a molecular weight of about 35 kDa was expressed in *E.coli* BL21 (DE3) and purified using the Ni^2+^-charged affinity columns. Before purification, the recombinant protein was confirmed to be soluble by ultrasonic treating BL21 (DE3) and SDS-PAGE of the supernatant and precipitate respectively according to His Bind Purification Kit manual (Novagen, Germany). The purified protein had the expected molecular weight (17.13 kDa as Ca-AIF1 and about 18 kDa as pET-32a tags) as detected by SDS-PAGE and later by western blot analysis using the prepared anti-CaAIF1 rabbit anti-serum (Figure 4A and B).

### Expression profile of Ca-AIF1 in the hemocyte monolayer after RLO/LPS challenge

**Figure 5:**
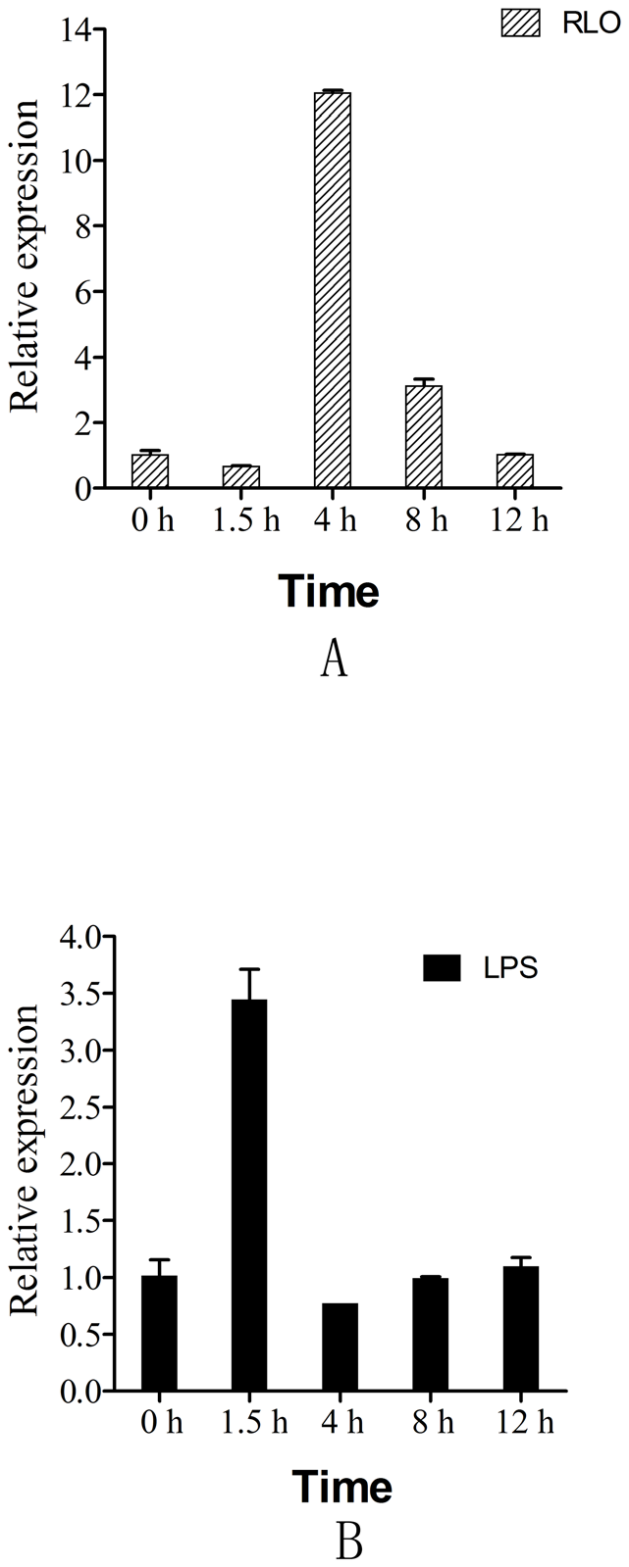
mRNA expression levels of Ca-AIF1 in hemocyte monolayers at different times after RLO/LPS incubation. Samples were collected at 0, 1.5, 4, 8 and 12(1 µl RLO/10^6^ hemocytes) or LPS (100 ng/ml) incubation. Expression levels were determined by real-time RT-PCR. The seawater-incubated groups were used as controls. Expression levels were assessed using 28SrDNA gene for normalization. *p≤0.05 when compared to control value. (A) RLO; (B) LPS.

A hemocyte monolayer was prepared and a real-time RT-PCR approach was used to analyze the mRNA expression profile of Ca-AIF1 in cells challenged with RLO/LPS. For the RLO challenge, the mRNA expression levels of Ca-AIF1 at 4 h and 8 h post-challenge were significantly increased (about 12.05 and 3.10 times compared to control groups, respectively), while the mRNA expression level of Ca-AIF1 was only significantly increased at 1.5 h post-challenge with LPS (about 3.44 times compared to control group). At other time points, the expression levels of Ca-AIF1 showed no statistically differences (p value >0.05) (Figure 5).

### The effects of Ca-AIF1 on expression levels of LITAF, TGFβ and MyD88 in oyster hemocyte

**Figure 6:**
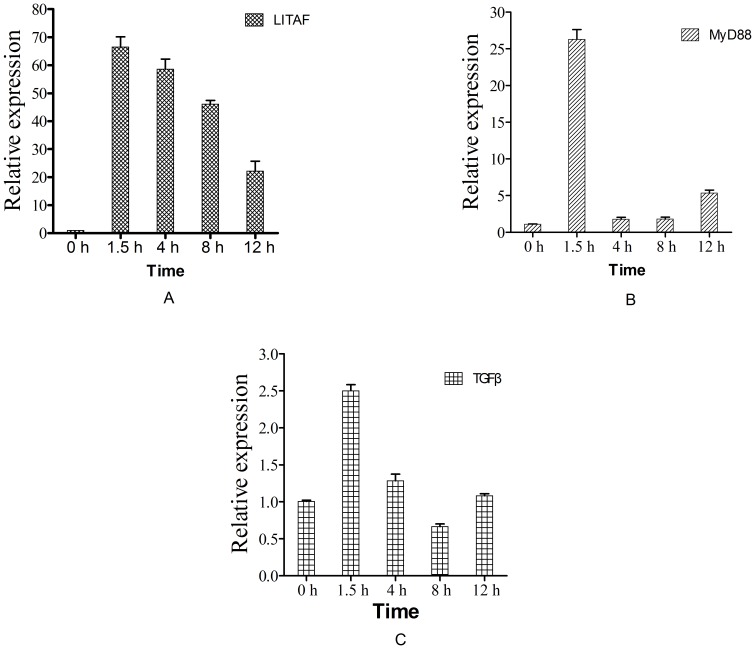
mRNA expression levels of LITAF,MyD88 and TGFβ in hemocyte monolayers. Samples were collected at different times after Ca-AIF1 protein (1 µg/ml) incubation. Expression levels were determined by real-time RT-PCR and seawater-incubated groups were used as controls. Expression levels were assessed using 28SrDNA gene for normalization. *p≤0.05 when compared to control value. (A) LITAF; (B) MyD88; (C) TGFβ.

We previously obtained the gene sequences of a series of immune-related genes from *C. ariakensis*. Here, in order to further investigate the functions of Ca-AIF1 in the oyster inflammation and immune response, we carried out real-time RT-PCR analysis to investigate the effects of Ca-AIF1 incubation *in vitro* on mRNA expression levels of immune-related genes LITAF, TGFβ and MyD88. The results showed that Ca-AIF1 treatment significantly up-regulates the expression levels of LITAF, MyD88 and TGFβ(Figure 6). The mRNA expression level of LITAF was significantly induced at all selected time points after Ca-AIF1 incubation *in vitro*. It reached the highest level (66.53-fold higher compared to the control) at 1.5 h post-incubation. After that time, the rates were 58.61, 46.12 and 22.18 times higher compared to the control group respectively, from 4–12 h after incubation. Similarly, the mRNA expression level of MyD88 was significantly induced at all selected time points after Ca-AIF1 incubation *in vitro*, but the up-regulation was lower than for LITAF. The highest level was 26.31-fold higher compared to the control group at 1.5 h post-incubation but the MyD88 mRNA expression level (5.38-fold higher) at 12 h post-incubation was higher than the levels at 4 h (1.82-fold) and 8 h (1.87-fold) post- incubation. Unlike LITAF and MyD88, the mRNA expression level of TGFβ was only up-regulated at 1.5 h post-incubation and about 2.50 times higher in contrast to the control group.

### The effect of anti-CaAIF1 RLO/LPS-induced LITAF expression

**Figure 7:**
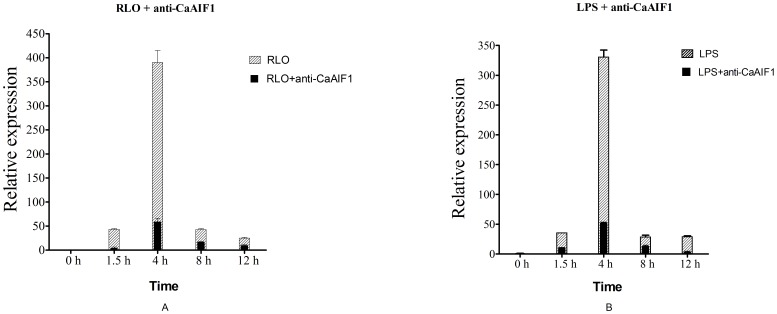
mRNA expression levels of LITAF in hemocyte monolayers. Samples were collected at different times after RLO (1 µl RLO/10^6^ hemocytes) + anti-CaAIF1 (1∶1000) and LPS (100 ng/ml) + anti-CaAIF1 (1∶1000) incubation and expression levels were determined by real-time RT-PCR. The seawater-incubated groups were used as controls. Expression levels were assessed using 28SrDNA gene for normalization. *p≤0.05 when compared to control value. Δ p≤0.05 when RLO/LPS + anti-CaAIF1 group value compared to RLO/LPS group value. (A) RLO + anti-CaAIF1; (B) LPS + anti-CaAIF1.

LITAF is an important transcription factor and believed to regulate the expression of inflammatory-related factors IL-1α, TNFα and IFN-γ [32], [33]. Challenge of RLO/LPS can induce the mRNA expression level of LITAF strongly. Therefore, in order to investigate the effect of the prepared Ca-AIF1 antibody on decreasing inflammatory reactions *in vitro*, we added prepared anti-CaAIF1 polyclonal antiserum to the RLO/LPS challenged hemocyte monolayer, and then performed real-time RT-PCR to analyze the mRNA expression level variation of LITAF at 1.5 h, 4 h, 8 h and 12 h post-treatment. The results show that the RLO/LPS challenge can up-regulate the expression level of LITAF in hemocytes, while administration of anti-CaAIF1 reduces this effect. The reduction appeared from 1.5 h to 12 h post-treatment (Figure 7).

When challenged with RLO, mRNA expression levels of LITAF are up-regulated about 43.32, 390.18, 42.95 and 25.27-times higher compared to the control group at 1.5 h, 4 h, 8 h and 12 h post-challenge, respectively. Administration of anti-CaAIF1 antibody significantly reduced the expression levels of LITAF to 4.95, 59.32, 17.45 and 10.32 fold higher relative to the control group at 1.5 h, 4 h, 8 h and 12 h post-challenge, respectively, with a reduction of about 89%, 85%, 59% and 59%, respectively (Figure 7A).

When challenged with LPS, mRNA expression levels of LITAF were up-regulated immediately to about 35.59, 330.81, 28.73 and 29.14 times higher compared to the control group at 1.5 h, 4 h, 8 h and 12 h post-challenge, respectively. Administration of anti-CaAIF1 significantly reduced the expression levels of LITAF to 10.34, 52.35, 13.43 and 3.63 times higher compared to the control group at 1.5 h, 4 h, 8 h and 12 h post-challenge, respectively, with a reduction of around 71%, 84%, 39% and 88%, respectively (Figure 7B).

### The effect of anti-CaAIF1 antibody on the reduction of RLO/LPS-induced hemocyte apoptosis and necrosis rates

To further analyze CaAIF1 function, we used flow cytometry to detect the apoptosis and necrosis rates of hemocytes *in vitro* treated with anti-CaAIF1 antibody. The results show that administration of anti-CaAIF1 can reduce the RLO/LPS-induced cell apoptosis and necrosis rates of hemocytes and increase cell survival.

**Figure 8:**
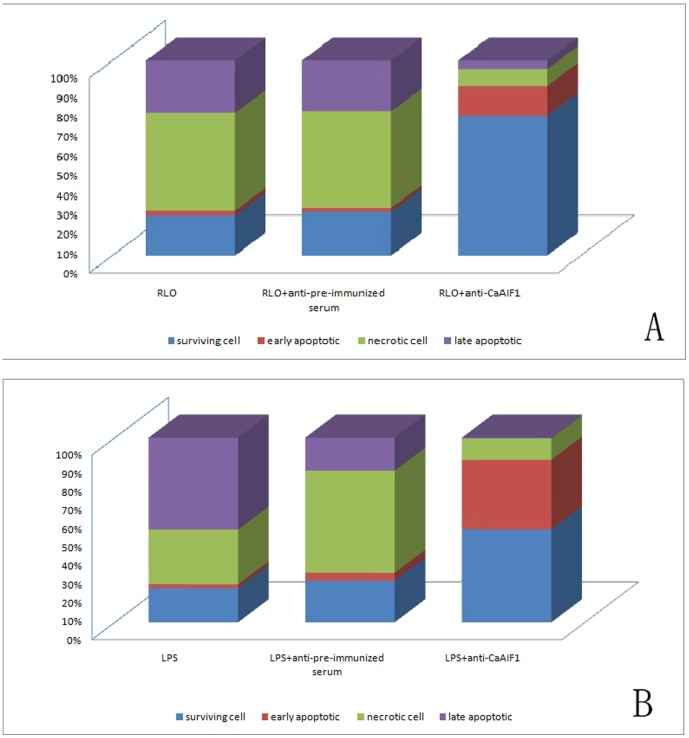
Flow cytometer analysis of hemocyte apoptosis and necrosis. Samples were collected 0 and 12/LPS (LPS, 100 ng/ml; RLO, 1 µl RLO/10^6^ hemocytes) + anti-CaAIF1 (1∶1000) incubation. The controls were hemocytes incubated with RLO/LPS and RLO/LPS + pre-immunized serum (1∶1000). (A) RLO challenged; (B) LPS challenged.

Compared to the two control groups (RLO challenged group and RLO + pre-immune serum challenged group), the percentage of late apoptotic cells in the RLO + anti-CaAIF1 antiserum (1∶1000) challenged group decreased by 22%, as did the percentage of necrotic cells (41%). The percentage of early apoptotic cells increased by 13%, and the percentage of surviving cells increased by 51% and 49%, respectively (Figure 8A). Compared to the two control groups (LPS challenged group and LPS + pre-immune serum challenged group), the percentage of late apoptotic cells in the LPS + anti-CaAIF1 antiserum (1∶1000) challenged group were decreased by 50% and 18% respectively, the percentage of necrotic cells by 18% and 44% respectively. The percentage of early apoptotic cells were increased by 35% and 33% respectively, and the percentage of surviving cells were increased by 32% and 28% respectively (Figure 8B).

## Discussion

Like other invertebrates, the oysters lack demonstrated acquired immunity and rely exclusively on the innate immune system to protect them against continuous threats from pathogens [34]. The identification and characterization of genes involved in the oyster immune response are critical to an understanding of immune defense mechanisms for disease control [2], [35], [36], [37], [38]. Herein, we report the molecular characterization, tissue distribution, transcriptional analysis and pro-inflammatory function analysis of the novel oyster AIF-1 together and exploring functions using a polyclonal antibody.

The AIF-1 is an IFN-γ-inducible Ca^2+^-binding EF-hand protein, which was first identified in rat cardiac allografts during chronic rejection [24], [26]. To date, several AIF-1 and AIF-1-like genes have been cloned from a wide range of species including invertebrates (e.g. sponge [39], clam [40], abalone [41]) and vertebrates (e.g. carp [42], red seabream [43], rat [26], human [44]). Deininger et al. [45] have described that AIF members encode proteins with a wide range of biologically active sites of motifs and suggested a systematic classification for AIF family proteins. AIFs were classified into four different subfamilies based on its amino acid length (147, 132, 93 and 57 amino acid residues, respectively) [45]. In this study, the identified Ca-AIF1 has 149 aa which is similar to the average size of AIF-1 family proteins. Sequence analysis shows that Ca-AIF1 possessed several characteristic motifs such as EF hand Ca^2+^ binding domains, casein kinase II phosphorylation sites, tyrosine kinase and protein kinase C phosphorylation sites and an N myristoylation site. Like in abalone and clam, the signal peptide sequence and NLS signal were not identified in Ca-AIF1, although sponge AIF-1 possessed a putative basic type NLS [39]. Furthermore, multiple sequence and phylogenetic analysis revealed that AIF-1 is a highly conserved gene among different Phyla of invertebrates and vertebrates, suggesting that AIF-1 is an essential molecular component functioning across species.

In mammals, the described expression of AIF-1 is mainly limited to cells of monocyte/macrophage lineage, however it is also expressed in muscle, liver, spleen and thymus in rat [44] and human [46]. In mollusks, AIF-1 transcripts are ubiquitously expressed in a variety of tissues with the highest expression level in hemocytes in the abalone and clam [40], [41]. Similarly, we observed that Ca-AIF1 transcript is ubiquitously expressed in all examined tissues including hemocytes, gills, mantle, digestive glands, gonads and adductor muscle, suggesting that Ca-AIF1 might participate in various biological processes in the oyster. Moreover, the highest expression level of Ca-AIF1 is observed in hemocytes that play a key role in oyster immunity.

AIF-1 was originally cloned from active macrophages in human and rat atherosclerotic allogenic heart grafts undergoing chronic transplant rejection [24], [26]. Studies of endomyocardial biopsy specimens from human heart transplants [25] and allogeneic grafts in sponge [39] also show that the expression of AIF-1 is elevated in allografts, suggesting that AIF-1 expression is induced in response to allogeneic antigens. Subsequent studies in mammals demonstrated that the expression of AIF-1 is increased in various host responses to inflammatory stimuli, suggesting that AIF-1 plays a role in inflammation [27], [46], [47], [48]. And some studies explained that AIF-1 could be secreted from immune cells in inflammatory conditions for functions. Brauner et al. [49] showed that CAPD patients with peritonitis produce high levels of daintain/AIF-1, which are secreted into the peritoneal fluid during infection and further demonstrated that daintain/AIF-1 increased significantly in the peritoneal fluid and supernatants of cultured THP-1 monocytes and macrophages in response to different live bacteria stimulation. Koshiba et al. [50] stated that AIF-1 protein was present in greater amounts in peritoneal fluid from patients with endometriosis than in women without it. Their further study showed that cultured peritoneal macrophages from endometriosis patients secreted more AIF-1 than those from unaffected women, and AIF-1 release can be stimulated by IL-1β and IFN-γ. Kimura et al. [51] demonstrated that AIF-1 was more strongly expressed in the synovial tissue of RA patients compared with the OA patients, also the levels of AIF-1 protein were higher in synovial fluid from patients with RA compared with patients with OA.

In aquatic animals, Miyata et al. [43] have shown that in the red sea bream AIF-1 transcripts are elevated in leukocytes from 3 to 24 h after LPS stimulation, implying that the AIF-1 in this fish might have a similar function in active leukocytes as does its homolog in mammals. In the abalone, both pathogenic challenge and tissue injury up-regulate the expression level of AIF-1 [41] and in the clam, the mRNA expression level of AIF-1 was first down–regulated, then increased in haemocytes after bacterial challenge [40], indicating that AIF-1 might be involved in the response to immune challenge in mollusks. In the present study, we prepared a hemocyte monolayer incubated with RLO and LPS. Real-time RT-PCR was used for investigating the temporal effect of RLO/LPS challenge on the transcriptional activity of Ca-AIF1. The result shows that RLO/LPS challenge can increase the mRNA expression level of AIF-1. Compared to LPS challenge, RLO challenge caused a later and longer elevation of AIF-1 transcription.

Watano et al. [27] reported that upon stimulation with bacterial lipopolysaccharide, transfected cells that overexpressed AIF-1 showed marked morphological change and produced significantly more IL-6, IL-10 and IL-12p40 compared to control cells, suggesting that AIF-1 plays an important role in cells of a monocyte/macrophage lineage upon stimulation with inflammatory stimuli by augmenting cytokine production. Liu et al. [52] described that daintain/AIF-1 promotes breast cancer proliferation by activating the NF-κB/cyclin D1 pathway and facilitates breast tumor growth in nude mice. Li et al. [53] further used a mimic tumor microenvironment by incubating breast cancer cells within the medium with or without daintain/AIF-1 polypeptide to evaluate cell migration and demonstrated that daintain/AIF-1 activates p38 MAPK pathways, contributes to the up-regulation of TNFα and consequently enhances breast cancer cell migration. Kimura et al. [51] used purified rAIF-1 to incubate human synoviocytes and PBMCs cells, and demonstrated that the IL-6 increased after stimulation for 24 h in a dose-dependent manner. And this AIF-induced IL-6 secretion can be inhibited by anti-AIF-1Ab. In addition, AIF-1 can stimulate the release of several other cytokines and growth factors including FGF-2, PDGF, TGFβ, G-CSF and MCP-1 [48], [54], [55].

In this study, we used purified recombinant Ca-AIF1 protein to incubate and stimulate the prepared oyster hemocyte monolayer, which is thought to be primarily immune cell, and real-time RT-PCR analysis was used to detect the expression variation of immune-related genes LITAF, TGFβ and MyD88 at the mRNA level. The results showed that Ca-AIF1 could significantly up-regulate the expression of LITAF, MyD88 and TGFβ. Among the three genes detected, LITAF is an important transcription factor and believed to regulate the expression of inflammatory-related factors IL-1α, TNFα and IFN-γ in mammals [32], [33]. TNFα and TGFβ participate in several biological processes such as cell differentiation and inflammatory reaction [56], [57]. MyD88 is the key downstream adaptor for most Toll-like receptors (TLRs) and interleukin-1 receptors (IL1Rs) [5], [58]. Ca-AIF1 is implicated in the regulation of the above immune-related or inflammatory-related factors, so we suggest that Ca-AIF1 has potential pro-inflammatory regulatory function and might take part in immune response for RLO infection or LPS-induced inflammation reaction through MyD88-mediating pathways.

To further investigate the immune and inflammatory functions of this protein, we produced its polyclonal anti-serum and designed experiments to study the effects of anti-CaAIF1 on restraining RLO/LPS-induced inflammatory reactions. We added anti-CaAIF1 polyclonal antiserum to the RLO/LPS challenged hemocyte monolayer, and performed real-time RT-PCR to observe prepared anti-CaAIF1 polyclonal antiserum function on reducing RLO/LPS-induced LITAF expression. Flow cytometry was used to analyze anti-CaAIF1 function on reducing RLO/LPS-induced cell apoptosis and necrosis of hemocytes. The results showed that administration of anti-CaAIF1 reduces RLO/LPS-induced up-regulation of LITAF from 1.5–12 h post-treatment. Also, administration of anti-CaAIF1 can reduce the RLO/LPS-induced cell apoptosis and necrosis and increase the cell survival rate of hemocytes. These results indicate that anti-CaAIF1 polyclonal antiserum can significantly inhibit the RLO-induced infection/LPS-induced inflammatory reaction. It has the potential to be used as anti-RLO or antibacterial components in the future.

In conclusion, we report the molecular characterization of a novel AIF-1 from oyster *C. ariakensis*, which is structurally related to the AIF-1 protein family. The mRNA expression levels of Ca-AIF1 were up-regulated after RLO/LPS challenged. Our experiments indicate that Ca-AIF1 functions in the regulation of the immune-related genes LITAF, MyD88 and TGFβ. Administration of anti-CaAIF1 antibody can effectively suppress RLO/LPS-induced LITAF up-regulation and RLO/LPS-induced necrosis and apoptosis of hemocytes. These results indicate that Ca-AIF1 functions as a pro-inflammatory cytokine and is a potential target for preventing and controlling RLO infection or LPS-induced inflammation.

## Supporting Information

Checklist S1 CONSORT checklist.
(DOC)
